# Probing optical anisotropy of nanometer-thin van der waals microcrystals by near-field imaging

**DOI:** 10.1038/s41467-017-01580-7

**Published:** 2017-11-13

**Authors:** Debo Hu, Xiaoxia Yang, Chi Li, Ruina Liu, Ziheng Yao, Hai Hu, Stephanie N. Gilbert Corder, Jianing Chen, Zhipei Sun, Mengkun Liu, Qing Dai

**Affiliations:** 1 Nanophotonics Research Division, CAS Center for Excellence in Nanoscience, National Center for Nanoscience and Technology, Beijing, 100190 China; 2 Department of Physics, Stony Brook University, Stony Brook, NY 11794 USA; 3 Institute of Physics, Chinese Academy of Science, Beijing, 100190 China; 4 Department of Electronics and Nanoengineering, Aalto University, Tietotie 3, FI-02150 Espoo, Finland

## Abstract

Most van der Waals crystals present highly anisotropic optical responses due to their strong in-plane covalent bonding and weak out-of-plane interactions. However, the determination of the polarization-dependent dielectric constants of van der Waals crystals remains a nontrivial task, since the size and dimension of the samples are often below or close to the diffraction limit of the probe light. In this work, we apply an optical nano-imaging technique to determine the anisotropic dielectric constants in representative van der Waals crystals. Through the study of both ordinary and extraordinary waveguide modes in real space, we are able to quantitatively determine the full dielectric tensors of nanometer-thin molybdenum disulfide and hexagonal boron nitride microcrystals, the most-promising van der Waals semiconductor and dielectric. Unlike traditional reflection-based methods, our measurements are reliable below the length scale of the free-space wavelength and reveal a universal route for characterizing low-dimensional crystals with high anisotropies.

## Abstract (web summary)

The optical response of van der Waals layered crystals is strongly anisotropic. Here, the authors develop a nano-imaging technique to determine the in-plane and out-of-plane components of the anisotropic dielectric tensors in MoS_2_ and hBN, two representative van der Waals crystals.

## Introduction

Symmetry breaking and intrinsic anisotropy are commonplace in low-dimensional materials^1^. This is especially the case in two-dimensional (2D) van der Waals (vdW) crystals^2^, where the strong in-plane covalent bonds and weak out-of-plane vdW forces naturally lead to highly anisotropic material properties. For example, the marked optical or electronic anisotropy has led to renowned investigations of hyperbolic dispersion in hexagonal boron nitride (h-BN)^3–7^, linear dichroism in black phosphorus^8–10^, spin-dependent relaxation in graphene^11–13^, and valley polarization in molybdenum disulfide (MoS_2_)^14,15^. By stacking 2D vdW crystals layer-by-layer into heterostructures (vdWHs)^16–19^, a series of novel optoelectronic and photonic applications have been demonstrated as well, including light-emitting diodes^20,21^, plasmonic waveguides^22–24^, and photodetectors^25^. Although in their experimental infancy^26^, these promising applications demand better identification of the anisotropic properties of various building blocks of vdWHs, to facilitate the rational design and optimization of vdWHs-based devices.

The optical anisotropy in vdW crystals, especially between the in-plane and out-of-plane directions, is challenging to measure. It is more so in the case of mechanically exfoliated crystals, which are known to possess superior crystalline qualities. The typical size of the exfoliated vdW crystals can be of the order of a few microns, which prohibits most of the common diffraction-limited characterization techniques such as edge reflection (requiring large sample thickness and surface area, ~mm^3^-scale volume at least; in addition, fine polished cross-sectional surface is required to measure the out-of-plane dielectric constant)^27^ and ellipsometry (requiring oblique incident angles and large sample area, ~100 × 55 μm^2^ at least)^28^. Therefore, it is a nontrivial task to obtain the intrinsic polarization-dependent optical properties of high-quality vdW crystals. A novel method is highly desired for evaluating and quantifying the optical anisotropy of nanometer-thin vdW microcrystals.

In this work, we elaborate on a method for characterizing the optical anisotropy of nanometer-thin vdW microcrystals. Using a scattering-type scanning near-field optical microscope (s-SNOM), the full dielectric tensor of vdW nanoflakes can be quantitatively extracted from real-space mapping of the ordinary and extraordinary waveguide modes. With this method, we report the first measurement of the dielectric tensor of MoS_2_ microcrystals (in-plane/out-of-plane permittivity is 20.25/9.61) in the near-infrared region (wavelength *λ* = 1530 nm). By extending the working wavelength to the visible region (wavelength *λ* = 632.8 nm), the optical anisotropy of h-BN can also be characterized (in-plane/out-of-plane permittivity is 5.33/2.99) and compared to the previous reported results. This work breaks the experimental bottleneck necessitating large-size samples in order to characterize the polarization-dependent optical properties of low-dimensional vdW crystals.

## Results

### Theoretical foundation for the method

Since MoS_2_ is a uniaxial vdW crystal with its optic axis *c* perpendicular to the basal plane (Supplementary Fig. 1), its relative dielectric tensor can be written as

1 $$\left\| \varepsilon \right\| = \left[ {\begin{array}{*{20}{l}} {{\varepsilon _ \bot }} \hfill & 0 \hfill & 0 \hfill \\ 0 \hfill & {{\varepsilon _ \bot }} \hfill & 0 \hfill \\ 0 \hfill & 0 \hfill & {{\varepsilon _\parallel }} \hfill \\ \end{array}} \right],$$

where *ε*_⊥_ is the in-plane relative dielectric constant (perpendicular to the optic axis), and *ε*_*‖*_ is the out-of-plane relative dielectric constant (parallel to the optic axis). In analogy with the ordinary and extraordinary rays in bulk anisotropic crystals^29^, it can be proven theoretically (Supplementary Note 1) that there are ordinary and extraordinary waveguide modes propagating in the anisotropic MoS_2_ nanoflakes. The eigenequations of the waveguide modes can be written as

2 $$\sqrt {{\varepsilon _ \bot }k_0^2 - \beta _{\rm{o}}^2} d = {\tan^{ - 1}}\left( {\frac{{\sqrt {\beta _{\rm{o}}^2 - k_0^2{\varepsilon _1}} }}{{\sqrt {{\varepsilon _ \bot }k_0^2 - \beta _{\rm{o}}^2} }}} \right) \\ + {\tan^{ - 1}}\left( {\frac{{\sqrt {\beta _{\rm{o}}^2 - k_0^2{\varepsilon _2}} }}{{\sqrt {{\varepsilon _ \bot }k_0^2 - \beta _{\rm{o}}^2} }}} \right) + m\pi $$

and

3 $$\sqrt {\frac{{{\varepsilon _ \bot }}}{{{\varepsilon _\parallel }}}} \sqrt {{\varepsilon _\parallel }k_0^2 - \beta _{\rm{e}}^2} d = {\tan ^{ - 1}}\left( {\frac{{\sqrt {\beta _{\rm{e}}^2 - k_0^2{\varepsilon _1}} {\varepsilon _ \bot }}}{{\sqrt {\frac{{{\varepsilon _ \bot }}}{{{\varepsilon _\parallel }}}} \sqrt {{\varepsilon _\parallel }k_0^2 - \beta _{\rm{e}}^2} {\varepsilon _1}}}} \right) \\ + {\tan ^{ - 1}}\left( {\frac{{\sqrt {\beta _{\rm{e}}^2 - k_0^2{\varepsilon _2}} {\varepsilon _ \bot }}}{{\sqrt {\frac{{{\varepsilon _ \bot }}}{{{\varepsilon _\parallel }}}} \sqrt {{\varepsilon _\parallel }k_0^2 - \beta _{\rm{e}}^2} {\varepsilon _2}}}} \right) + n\pi ,$$

respectively (Supplementary Note 2). In Eqs. 2 and 3, ${k_0} = 2\pi /\lambda$ is the free-space wavevector; *β*_o_ and *β*_e_ are the in-plane wavevectors for ordinary and extraordinary waveguide modes, respectively; *d* is the thickness of MoS_2_ nanoflakes; *ε*_1_ and *ε*_2_ are relative dielectric constants of the isotropic superstrate and substrate, respectively; and *m* and *n* are the order numbers (non-negative integers) of ordinary and extraordinary waveguide modes, respectively. According to the two transcendental equations above, the ordinary waveguide modes are transverse electric (TE) polarized, and their in-plane wavevectors are only related to the in-plane relative dielectric constant of MoS_2_; the extraordinary waveguide modes are transverse magnetic (TM) polarized, and their in-plane wavevectors are related to the in-plane and out-of-plane relative dielectric constants. Therefore, once the in-plane wavevectors of both the ordinary and extraordinary waveguide modes are determined for at least two MoS_2_ nanoflakes with different thicknesses, the in/out-of-plane relative dielectric constants can be found explicitly utilizing Eqs. 2 and 3.

### Experimental verification of the imaging principle

**Fig. 1:**
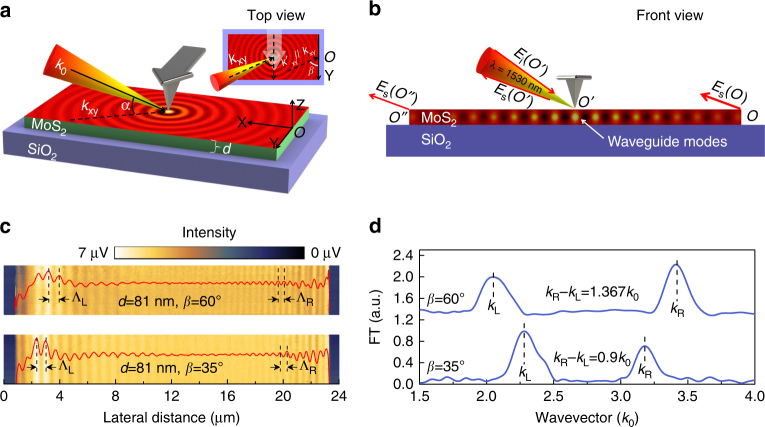
Schematics of the experimental setup and the near-field imaging principle. **a** Three-dimensional schematic of the near-field setup. The sharp edges of MoS_2_ nanoflakes are aligned to the *Y* axis and the s-SNOM tip scans along the *X* axis. Inset is the top view of the experimental setup. *α* is the angle between the illumination wavevector *k*_0_ and its projection in the *X*–*Y* plane *k*_*xy*_, *β* is the angle between *k*_*xy*_ and the investigated sample edges. **b** Front view of the experimental setup. The tip-launched waveguide modes are scattered into free space at the sample edges and interfere with the tip-scattered light at the photodetector. **c** Near-field images and real-space fringe profiles of the 81-nm-thick MoS_2_ sample with *β* = 60°and *β* = 35°, respectively. Λ_L_ is the fringe spacing at the left half of the near-field images while Λ_R_ is that at the right half. **d** Momentum–space spectra of the fringe profiles in **c**, the difference between the left and right side apparent wavevectors decreases with the reduction of *β*

In this work, the atomic force microscope (AFM)-based s-SNOM with nanoscale spatial resolution is employed to simultaneously acquire both the sample thickness and the in-plane wavevectors required by Eqs. 2 and 3. The experimental setup and imaging principle are illustrated in Fig. 1a, b.

As shown in Fig. 1a, the MoS_2_ nanoflakes on SiO_2_/Si substrates are preferentially oriented under the s-SNOM such that the sharp edges of the nanoflakes (coincident with the *Y* axis) are parallel to the AFM cantilever. The AFM cantilever can be used as a reference to infer geometric factors: the angle between the illumination wavevector *k*_0_ and its projection *k*_*xy*_ in the *X*–*Y* plane (coincident with the sample surface) is *α* = 38°; the angle between *k*_*xy*_ and the investigated sample edge is *β* = 60° (indicated in the top view). The near-infrared laser at *λ* = 1530 nm with 3 μm spot size was focused onto the apex of the s-SNOM tip to excite both ordinary and extraordinary waveguide modes in the MoS_2_ nanoflakes. These modes can propagate in the MoS_2_ nanoflakes as cylindrical waves, get scattered into the far field as free-space light at the sample edges or, in principle, back reflected. Because their in-plane wavevectors are far smaller than those of the graphene surface plasmon polaritons (SPPs)^30,31^ and h-BN surface phonon polaritons (SPhPs)^32^, back-reflection of the waveguide modes at the sample edges is fairly insufficient compared to previously studied cases^33,34^. Therefore, the acquired s-SNOM images are dominated by the interference fringe patterns formed between the tip-scattered light and the edge-scattered light as illustrated in Fig. 1b, implying significant dependence on the sample edge orientation. This makes the imaging principle of the waveguide modes different from those for graphene SPPs and h-BN SPhPs, where the resulting s-SNOM images are standing wave patterns formed by the incident and reflected surface waves, exhibiting no dependence on the sample edge orientation. Due to the asymmetry introduced by the incident angle, the optical path difference (OPD) between the tip-scattered light *E*_s_(*O*′) and the left side edge-scattered light *E*_s_(*O*″) is different from that between *E*_s_(*O*′) and the right side edge-scattered light *E*_s_(*O*). Note that the edge-scattered light in the vicinity of *O* and *O*″ has the major contribution to the near-field contrast, since the net scattering from all the other points will be diminished due to the in-plane symmetry and destructive interference. Therefore, the fringe spacing at the left half of the resulting s-SNOM image Λ_L_ is different from Λ_R_ at the right half. Based on the simple geometry in Fig. 1a, the genuine in-plane wavevectors of ordinary and extraordinary waveguide modes of MoS_2_ nanoflakes, *β*_o,e_, can be extracted either from the left side apparent wavevector *k*_L_ = 2*π*/Λ_L_ as

4 $${\beta _{{\rm{o,e}}}} = \frac{{2\pi }}{{{\Lambda _{\rm{L}}}}} + {k_0}\,\cos \,\alpha \,\sin \,\beta ,$$

or from the right side apparent wavevector *k*_R_ = 2*π*/Λ_R_ as

5 $${\beta _{{\rm{o,e}}}} = \frac{{2\pi }}{{{\Lambda _{\rm{R}}}}} - {k_0}\,\cos \,\alpha \,\sin \,\beta .$$

We first demonstrate the validity of the above imaging principle experimentally with an 81-nm-thick MoS_2_ sample placed in the same orientation as in Fig. 1a, i.e., *β* = 60° (see Supplementary Fig. 2 for AFM images and height profiles). The real-space s-SNOM image together with the corresponding fringe profile is shown in the upper panel of Fig. 1c. As expected, the fringe spacings are different at the opposite edges: the spacing at the left edge is 747 nm (corresponding to an apparent in-plane wavevector *k*_L_ = 2.048*k*_0_), while at the right it is 448 nm (*k*_R_ = 3.415*k*_0_). The wavevector information can be represented more clearly in momentum space as shown in the upper panel of Fig. 1d by imposing a Fourier transform (FT) on the real-space fringe profile (see Supplementary Fig. 3 for detailed data-processing method). The frequency peaks at the local maxima correspond to the apparent in-plane wavevectors *k*_L_ and *k*_R_ derived from the spatial domain, and their frequency difference coincides with the theoretical value 1.365*k*_0_ given by Eqs. 4 and 5. When *β* is reduced to 35° via sample rotation, a smaller (larger) fringe spacing at the left (right) edge is observed (lower panel of Fig. 1c) and the frequency difference between *k*_L_ and *k*_R_ is decreased (lower panel of Fig. 1d). More importantly, experiments at different *β*s produce the same in-plane wavevector 2.735*k*_0_ for the waveguide mode in the same sample, validating the rigorous parameter extraction procedure. Therefore, we have established a self-consistent method to measure the in-plane wavevectors of waveguide modes propagating in the nanometer-thin vdW microcrystals using s-SNOM.

### Extraction of the dielectric tensor from real-space images

**Fig. 2:**
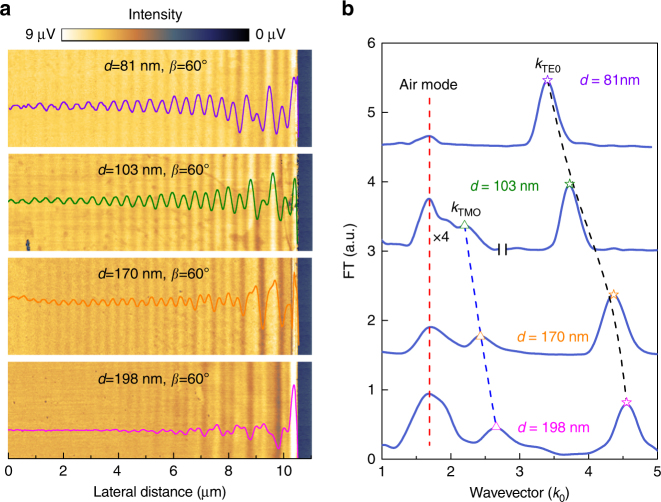
Experimental results. **a** Near-field images and real-space fringe profiles of MoS_2_ samples with different thicknesses. **b** Momentum–space spectra of fringe profiles in **a**. In the experiments, the MoS_2_ nanoflakes are all placed in the same orientation as in Fig. 1a. The low-frequency peaks in **b** showing no thickness dependence are the trivial air modes. Note that in **b** the left part of the second spectrum (*d* = 103 nm) is multiplied by four

Since the MoS_2_ nanoflake in Fig. 1c is relatively thin, it supports only one waveguide mode—the fundamental (*m* = 0) ordinary mode, i.e. the TE_0_ mode. For thicker MoS_2_ flakes, two or more distinct modes can be observed. For example, in a 103-nm-thick MoS_2_ flake, a fundamental extraordinary mode (TM_0_ mode) and a trivial low-frequency air mode are evident in addition to the TE_0_ mode (Fig. 2). The wavevector of the trivial air mode equals ${k_0}(1 + \cos \alpha \sin \beta )$ and does not shift with the increasing sample thickness. The apparent wavevectors of the TM_0_ and TE_0_ modes are thickness-dependent and shift towards higher frequencies, eventually separating themselves from the air modes as the sample thickness increases above ~150 nm, as shown in Fig. 2b.

The TE_0_ mode provides an in-plane wavevector ${k_{\rm{R}}}\! -\! {k_0}\,\cos \,\alpha \,\sin \,\beta$. By substituting the two sets of experimental data (*d*_1_ = 81 nm, *β*_o1_ = 2.735*k*_0_; *d*_2_ = 103 nm, *β*_o2_ = 3.06*k*_0_) into Eq. 2, we get the in-plane dielectric constant *ε*_⊥_ = 20.25 and the TE mode order number *m* = 0. Similarly, the in-plane wavevectors of the TM_0_ modes are determined to be 1.733*k*_0_ and 2.007*k*_0_ for the 170-nm-thick and 198-nm-thick samples, respectively. By substituting these two sets of data (*d*_3_ = 170 nm, *β*_e1_ = 1.733*k*_0_; *d*_4_ = 198 nm, *β*_e2_ = 2.007*k*_0_) into Eq. 3, one can get the out-of-plane dielectric constant ${\varepsilon _\parallel } = 9.61$ and the TM mode order number *n* = 0. Therefore, the relative dielectric tensor of MoS_2_ governing its optical anisotropy at the important optical communication wavelength 1530 nm can be quantitatively determined to be

6 $$\left\| \varepsilon \right\| = \left[ {\begin{array}{*{20}{l}} {20.25} \hfill & 0 \hfill & 0 \hfill \\ 0 \hfill & {20.25} \hfill & 0 \hfill \\ 0 \hfill & 0 \hfill & {9.61} \hfill \\ \end{array}} \right],$$

which clearly demonstrates that the MoS_2_ nanoflakes are indeed negative crystals since the extraordinary index of refraction (${n_{\rm{e}}} = \sqrt {{\varepsilon _\parallel }} = 3.1$) is less than the ordinary one (${n_{\rm{o}}} = \sqrt {{\varepsilon _ \bot }} = 4.5$). Note that we chose to calculate the out-of-plane (in-plane) dielectric constant using the thicker (thinner) samples because the corresponding wavevectors are the most prominent with those thicknesses, yielding a more accurate parameter extraction. The experimentally obtained dielectric tensor of MoS_2_ is in very good agreement with theoretical values by first principle calculations (${\varepsilon _ \bot } = 16.8,{\rm{ }}{\varepsilon _\parallel } = 9.0$, note that these are stationary values calculated at long wavelength limit; at 1530 nm, the values are indeed expected to be larger)^35,36^.

## Discussion

As demonstrated above, to disentangle the in-plane and the out-of-plane dielectric constants and quantify the full dielectric tensor of the investigated vdW microcrystals, one has to image both TE-polarized ordinary and TM-polarized extraordinary waveguide modes. The aperture-type SNOM (a-SNOM) has been widely used in the waveguide mode imaging as demonstrated in the previous works^37,38^. Compared to a-SNOM, we suggest using s-SNOM to study the anisotropy of low-dimensional vdW microcrystals due to its wavelength-independent high-spatial resolution (~10 nm) and ultra-broadband compatibility. However, imaging the TE modes with s-SNOM is not routine work since it has been long believed that s-SNOM can only effectively excite and pick up the TM-polarized near-field signals due to the elongated tip geometry perpendicular to the sample surface. Nevertheless, we managed to image the TE-polarized waveguide modes for the first time. The imaging capability of the s-SNOM for TE-polarized modes remained undiscovered mostly because the s-SNOM has been applied mainly in the mid-infrared region where the TM-polarized field dominates the near-field scattering signal^39^. In addition, the surface or waveguide modes such as graphene SPPs^30,31^ and h-BN SPhPs^32^ investigated in previous s-SNOM experiments are exclusively TM polarized. The s-SNOM imaging of TE-polarized waveguide modes in this work can be attributed to the reduced working wavelength in the visible and near-infrared frequency ranges, where the tip geometry perpendicular to the sample surface is less important for determining the scattered near-field signal (in contrast, a spheroidal finite-dipole description is required in the mid-infrared as a result of the long working wavelength)^40^.

**Fig. 3:**
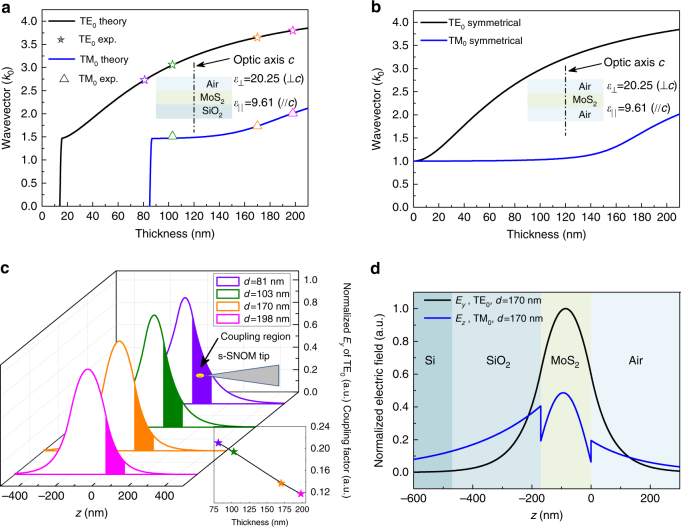
Analyses of experimental results. **a** Theoretical and experimental thickness dispersions of the fundamental ordinary (TE_0_) and extraordinary (TM_0_) waveguide modes in the air–MoS_2_–SiO_2_ three-layer waveguide, the superstrate air, and the substrate SiO_2_ are assumed to be semi-infinite in the calculations. **b** Theoretical thickness dispersions of the fundamental ordinary (TE_0_) and extraordinary (TM_0_) waveguide modes in free-standing MoS_2_ nanoflakes. **c** Evolution of mode profiles associated with the fundamental ordinary waveguide mode (TE_0_), the inset shows a decreasing coupling factor between the tip-induced hot spot and the waveguide mode with increasing sample thickness. We assume the interval 0 nm ≤ *z* ≤ 100 nm to be the efficient coupling region since the tip-tapping amplitude is set to 50 nm in the experiments. **d** Normalized mode profiles of the fundamental ordinary (TE_0_) and extraordinary (TM_0_) waveguide modes for the 170-nm-thick MoS_2_ sample indicate that the extraordinary mode retains stronger electric field at the virtual SiO_2_/Si interface and tends to leak out through the SiO_2_ layer. The calculations in **c** and **d** use the same air–MoS_2_–SiO_2_ three-layer waveguide model as in **a**

We calculated the thickness dispersion of the fundamental ordinary (TE_0_) and extraordinary (TM_0_) waveguide modes in the air–MoS_2_–SiO_2_ three-layer waveguide using Eqs. 2 and 3 by assuming that the superstrate air and the substrate SiO_2_ are both semi-infinite and taking their isotropic dielectric constants to be 1.00 and 2.15 at the 1530 nm wavelength^41^, respectively. The calculation results shown in Fig. 3a agree well with the in-plane wavevectors of the fundamental ordinary waveguide mode (TE_0_) in the 170-nm-thick and 198-nm-thick MoS_2_ samples (extracted from Fig. 2b). The slight deviation between the experimental and the simulated wavevector values for the 103-nm-thick sample is caused by the uncertainty in reading the position of the TM_0_ peak in Fig. 2b due to the air–TM_0_ modes overlapping mentioned above. Figure 3a indicates that the cutoff thicknesses for TE_0_ and TM_0_ modes in the air–MoS_2_–SiO_2_ asymmetrical waveguide are about 15 and 85 nm, respectively. Waveguides with MoS_2_ thickness larger than 85 nm can support both TE_0_ and TM_0_ modes; waveguides with MoS_2_ thickness in the interval between 15 and 85 nm can only support the TE_0_ mode, and when the MoS_2_ layer is thinner than 15 nm it cannot support any mode. The theoretically predicted cutoff of the fundamental extraordinary waveguide mode below the thickness of 85 nm explains the observed single-mode behavior of the 81-nm-thick sample shown in Fig. 2b. Thus, all the experimental results are in good agreement with each other in the framework of anisotropic waveguide theory.

The cutoff behaviors of the ordinary and extraordinary modes in asymmetrical waveguides (superstrate and substrate are of different dielectric constants, *ε*_1_ ≠ *ε*_2_) seemingly set a lower limit for the sample thickness we can investigate (Fig. 3a). However, the theoretical calculations demonstrate that the cutoff thicknesses of the fundamental modes decrease with the increasing degree of symmetry of the MoS_2_ waveguide (Supplementary Fig. 4); when the waveguide is perfectly symmetrical (*ε*_1_ = *ε*_2_) the fundamental modes do not cut off. Thus, by suspending the samples to eliminate the asymmetry, we can reduce this cutoff thickness for the fundamental waveguide modes. As shown in Fig. 3b, the in-plane wavevectors of both the ordinary and extraordinary fundamental modes approach the free-space wavevector asymptotically with the decreasing sample thickness, thus probing the optical anisotropies of few-layer or even monolayer samples utilizing our method is possible if the unwanted air mode can be suppressed. This is indeed probable since the air mode becomes weaker with the decreasing sample thickness as shown in Fig. 2b.

The contrast of the near-field images also has strong sample thickness dependence and is expected to fade out completely for samples much thicker than 200 nm (Figs. 2a and 3c). With increasing MoS_2_ sample thickness, the normalized electric field profile (Supplementary Note 3) of the fundamental ordinary mode shifts into the substrate, coupling much less with the s-SNOM tip-induced hot spot at the sample surface (see inset of Fig. 3c). This results in a decreased excitation efficiency of the waveguide mode and subsequent loss of interference visibility (i.e., image contrast). This fringe visibility of the lower order modes sets an upper limit for the sample thickness. For high-order modes, however, the evanescent fields extend much further out from the sample surface, leading to higher excitation efficiencies, and therefore enhanced interference visibilities with thicker samples (Supplementary Figs. 5 and 6). Generally speaking, the number of waveguide modes increases with the thickness of the MoS_2_ layer. For a waveguide with a 1000-nm-thick MoS_2_ layer, there are five TE modes (*m* = 0–4) and five TM modes (*n* = 0–4) (Supplementary Fig. 7) available for near-field imaging. Therefore, the sample thickness in our method is only limited by the maximum height measurement range of the AFM embedded in our s-SNOM (~1000 nm).

The last feature that cannot be overlooked in our experimental results is the unbalanced mode strength ratio between the fundamental ordinary and extraordinary modes in the momentum–space spectra as shown in Fig. 2b. To explain this phenomenon, we have to take the finite SiO_2_ substrate thickness (typical value 300 nm) into consideration because the waveguide modes tend to leak out through the SiO_2_ layer into the high refractive index Si layer below. As shown in Fig. 3d, the extraordinary mode (blue curve) in the 170-nm-thick MoS_2_ sample retains a stronger electric field at the virtual SiO_2_/Si interface than the ordinary mode (black curve), and hence experiences much higher dissipation during propagation^42^, which manifests as a low and broad peak in the momentum space. Therefore, we can reduce the transmission loss of the extraordinary mode by increasing the thickness of the SiO_2_ layer, which narrows its corresponding peak in the momentum space. This allows a more accurate determination of the out-of-plane dielectric constant by reducing the uncertainty in the peak position fitting procedure.

**Fig. 4:**
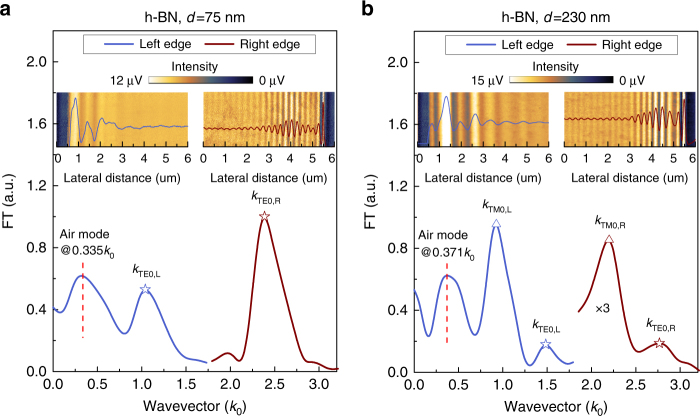
Probing optical anisotropy of h-BN in the visible region. Experimental results for **a** 75-nm-thick and **b** 230-nm-thick h-BN samples. In **a**, the h-BN nanoflake was placed in the same orientation as in Fig. 1a; in **b**, the two opposite edges are not exactly parallel and there are small angles between the edges and the direction of the s-SNOM tip cantilever as shown in Supplementary Fig. 9b. There is a small frequency difference between the air modes in **a** and **b** because of the different *β* angles shown in Supplementary Fig. 9. Note that in **b** the spectrum taken at the right edge is multiplied by three

The application of our method can also be validated for other vdW crystals as long as their transparent or low-loss frequency windows are known. For example, optical anisotropy of h-BN at the wavelength 632.8 nm has been investigated. Two h-BN samples with the thicknesses 75 and 230 nm are analyzed as shown in Fig. 4a, b, respectively (see also Supplementary Figs. 8 and 9). As shown in Fig. 4a, Fourier analysis of the left half of the fringe profile produces an air mode located at 0.335*k*_0_, indicating a different incident angle *α* = 39.8° for the visible laser from the one for the near-infrared laser (38°), this is quite reasonable since the visible and the near-infrared lasers cannot be in perfect alignment in the s-SNOM. Analysis of the other two peaks (TE_0_) in Fig. 4a using Eqs. 4 and 5 produces the same in-plane wavevector (1.68*k*_0_) for the fundamental ordinary mode propagating in the 75-nm-thick h-BN sample. Taking the small incline angles of the sample edges shown in Supplementary Fig. 9 into consideration, we can derive the in-plane wavevectors from Fig. 4b for the fundamental ordinary and extraordinary waveguide modes propagating in the 230-nm-thick h-BN sample (2.113*k*_0_ and 1.556*k*_0_, respectively) as well. Thus, the in-plane and out-of-plane relative dielectric constants of h-BN at 632.8 nm are determined to be 5.33 and 2.99, respectively, corresponding to an ordinary refractive index *n*_o_ = 2.31 and an extraordinary refractive index *n*_e_ = 1.73, respectively. The experimentally obtained refractive indices are slightly larger than those previously reported in artificially synthesized BN sample (*n*_o_ = 2.13, *n*_e_ = 1.65)^43^, probably because the polycrystalline and porous structure of the artificial BN sample used in the previous work tends to lower the refractive indices^44^ as compared with the intrinsic ones of the mechanically cleaved monocrystals used in this work.

The investigated MoS_2_ and h-BN samples in this work are uniaxial crystals. Nevertheless, this method can be applied to the much more complicated biaxial vdW crystals. To this end, one has to determine the two in-plane principal axes for the biaxial crystals by other techniques like Raman spectroscopy^45^ and second-harmonic generation (SHG)^46^. Waveguide mode imaging can be performed at the two sharp edges perpendicular to the two principal axes, naturally formed while crystal growth or machined using microfabrication techniques. The in-plane dielectric constant associated with each principal axis can be extracted following the same procedure used in the uniaxial vdW crystals characterization.

When properly mapped and characterized, the waveguide modes propagating in 2D materials can be a convenient way to determine the in-plane and out-of-plane dielectric constants. By employing near-field scanning methods, our work overcomes the challenge of measuring small-size samples of vdW crystals and provides two specific yet universally relatable examples in MoS_2_ and h-BN. The variations of the current method can lead to practical solutions for probing samples of in-plane anisotropy and few-layer or monolayer thickness. Further investigations will allow us to address important material properties at the nanoscale, such as the local dielectric properties around crystalline defects or the sub-wavelength polaritonic interactions in anisotropic nano-devices.

## Methods

### Sample preparation

Silicon wafers with 300-nm-thick SiO_2_ top layer were used as substrates for all the samples. The MoS_2_ and h-BN microcrystals of various thicknesses were exfoliated from bulk samples.

### Near-field optical measurement

The nano-imaging experiments described in the main text were performed using a commercial s-SNOM (www.neaspec.com). The s-SNOM is based on a tapping-mode AFM illuminated by monochromatic lasers of the wavelength 1530 or 632.8 nm (www.toptica.com). The near-field images were registered by pseudo-heterodyne interferometric detection module with tip-tapping frequency around 270 kHz, the tip-tapping amplitudes are 50 nm for the 1530-nm-wavelength experiments and 30 nm for the 632.8-nm-wavelength experiments. By demodulating the optical signal at the third order harmonic of the tip-tapping frequency, the noise from the background and stray light can be greatly suppressed. The spot sizes of the visible (632.8 nm) and near-infrared (1530 nm) beam at the focus under the AFM tip are ~1.5 and 3 μm, respectively, which are in favor of the tip-launching and edge-scattering detection scheme proposed in this research. Although there are certain areas near the edges where edge-launched waveguide modes exist, we can remove this edge effect in the data processing by windowing the real-space fringe profiles in the Fourier transform.

### Data availability

The data that support the findings of this study are available from the corresponding authors upon reasonable request.

## Electronic supplementary material

Supplementary Information
