# Supplementary material for: Probing optical anisotropy of nanometer-thin van der waals microcrystals by near-field imaging
Source: Nat Commun. 2017 Nov 13;8:1471. doi: 10.1038/s41467-017-01580-7 (PMC5684389; doi:10.1038/s41467-017-01580-7)
Supplement: Supplementary file 1 — Supplementary Information [file 41467_2017_1580_MOESM1_ESM.pdf]

Supplementary Note 1: Polarization States of Waves in Anisotropic Media

The wave equation in a homogeneous non-magnetic anisotropic medium is[1]

$$\nabla^2 \mathbf{E} + k_0^2 \|\varepsilon\| \mathbf{E} = \nabla(\nabla \cdot \mathbf{E}), \tag{1}$$

where $\|\varepsilon\|$ is the relative dielectric tensor. In the coordinate system aligned to the optic axis of a uniaxial anisotropic crystal, $\|\varepsilon\|$ can be expressed as

$$\|\varepsilon\| = \begin{bmatrix} \varepsilon_\perp & 0 & 0 \\ 0 & \varepsilon_\perp & 0 \\ 0 & 0 & \varepsilon_\parallel \end{bmatrix}, \tag{2}$$

where $\varepsilon_\perp$ is the relative dielectric constant perpendicular to the optic axis, and $\varepsilon_\parallel$ is the relative dielectric constant parallel to the optic axis.

Supplementary Equation (1) actually contains three equations, one for each dimension. Explicitly, these equations can be expressed in the matrix form as

$$\begin{bmatrix} k_y^2 + k_z^2 - \varepsilon_\perp k_0^2 & -k_x k_y & -k_x k_z \\ -k_y k_x & k_x^2 + k_z^2 - \varepsilon_\perp k_0^2 & -k_y k_z \\ -k_z k_x & -k_z k_y & k_x^2 + k_y^2 - \varepsilon_\parallel k_0^2 \end{bmatrix} \begin{bmatrix} E_x \\ E_y \\ E_z \end{bmatrix} = 0 \tag{3}$$

where $\mathbf{k} = (k_x, k_y, k_z)$ is the wavevector and $\mathbf{E} = (E_x, E_y, E_z)$ is the electric field. For a homogeneous wave in the Y direction, we have $k_y = 0$, then Supplementary Equation (3) can be reduced to

$$\begin{bmatrix} k_z^2 - \varepsilon_\perp k_0^2 & 0 & -k_x k_z \\ 0 & k_x^2 + k_z^2 - \varepsilon_\perp k_0^2 & 0 \\ -k_z k_x & 0 & k_x^2 - \varepsilon_\parallel k_0^2 \end{bmatrix} \begin{bmatrix} E_x \\ E_y \\ E_z \end{bmatrix} = 0. \tag{4}$$

For Supplementary Equation (4) to have a non-trivial solution (i.e. non-zero electric field), the determinant of the matrix must be zero:

$$\begin{vmatrix} k_z^2-\varepsilon_\perp k_0^2 & 0 & -k_x k_z \\ 0 & k_x^2+k_z^2-\varepsilon_\perp k_0^2 & 0 \\ -k_z k_x & 0 & k_x^2-\varepsilon_\parallel k_0^2 \end{vmatrix} = 0. \quad (5)$$

Solving Supplementary Equation (5) we get two sets of solutions:

$$k_{z1}=\sqrt{\varepsilon_\perp k_0^2-k_x^2},\ \mathbf{E}_{\mathrm{TE}}=\begin{bmatrix}0\\E_y\\0\end{bmatrix} \quad (6)$$

corresponding to the transverse electric (TE) polarized ordinary wave, and

$$k_{z2}=\sqrt{\frac{\varepsilon_\perp}{\varepsilon_\parallel}}\sqrt{\varepsilon_\parallel k_0^2-k_x^2},\ \mathbf{E}_{\mathrm{TM}}=\begin{bmatrix}E_x\\0\\E_z\end{bmatrix}. \quad (7)$$

corresponding to the transverse magnetic (TM) polarized extraordinary wave.

Supplementary Note 2: Eigenequations for Anisotropic Planar Waveguide

A three layer planar waveguide composed of an isotropic semi-infinite superstrate (ε_1), an anisotropic guiding layer ($\|\varepsilon\|$) with the thickness d, and an isotropic semi-infinite substrate (ε_2) is shown in Supplementary Fig. 1. The optic axis of the guiding layer is parallel to the Z axis.

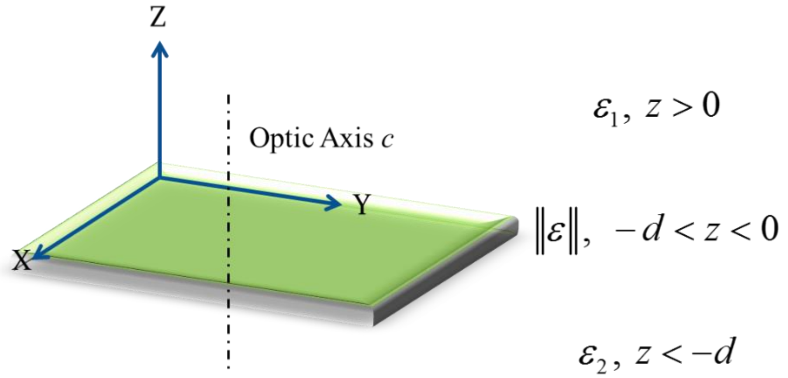

Supplementary Figure 1 | Schematic of a three-layer planar waveguide. The superstrate and substrate are both semi-infinite and optically isotropic. The optic axis of the anisotropic guiding layer is perpendicular to the basal plane.

For a waveguide mode (ordinary or extraordinary) propagating in the X direction in the three-layer planar waveguide shown in Supplementary Fig. 1, the electric field can be expressed as

$$\begin{cases} \mathbf{E}_1(\mathbf{r},t) = \mathbf{E}_1^0 e^{-\alpha_1 z} e^{i(\beta x-\omega t)} = (E_{1x}^0, E_{1y}^0, E_{1z}^0) e^{-\alpha_1 z} e^{i(\beta x-\omega t)} \\ \mathbf{E}_{\mathrm{g}}(\mathbf{r},t) = \mathbf{E}_{\mathrm{g}}^0 \cos(\alpha_{\mathrm{g}} z+\varphi) e^{i(\beta x-\omega t)} = (E_{\mathrm{g}x}^0, E_{\mathrm{g}y}^0, E_{\mathrm{g}z}^0) \cos(\alpha_{\mathrm{g}} z+\varphi) e^{i(\beta x-\omega t)} \\ \mathbf{E}_2(\mathbf{r},t) = \mathbf{E}_2^0 e^{\alpha_2 (z+d)} e^{i(\beta x-\omega t)} = (E_{2x}^0, E_{2y}^0, E_{2z}^0) e^{\alpha_2 (z+d)} e^{i(\beta x-\omega t)} \end{cases}, \tag{8}$$

where $\beta = k_x$ is the propagation constant of the waveguide mode (not to be confused with the angle in Figure 1 in the main text), $\alpha_{\mathrm{g}} = k_z$.

For the electric fields in the isotropic superstrate and substrate, Supplementary Equation (1) can be reduced to

$$\nabla^2 \mathbf{E} + k_0^2 \varepsilon_{1,2} \mathbf{E} = 0 . \tag{9}$$

Substituting $\mathbf{E}_1$ and $\mathbf{E}_2$ into Supplementary Equation (9), we get

$$\begin{cases} \alpha_1^2 = \beta^2 - k_0^2 \varepsilon_1 \\ \alpha_2^2 = \beta^2 - k_0^2 \varepsilon_2 \end{cases}. \tag{10}$$

Imposing Gauss's law $\nabla \cdot \left(\varepsilon_{1,2} \mathbf{E} \right) = 0$ on $\mathbf{E}_1$ and $\mathbf{E}_2$, we get

$$\begin{cases} \mathbf{E}_1(\mathbf{r},t) = (E_{1x}^0, E_{1y}^0, \dfrac{i\beta}{\alpha_1} E_{1x}^0) e^{-\alpha_1 z} e^{i(\beta x-\omega t)} \\ \mathbf{E}_2(\mathbf{r},t) = (E_{2x}^0, E_{2y}^0, \dfrac{-i\beta}{\alpha_2} E_{2x}^0) e^{\alpha_2 (z+d)} e^{i(\beta x-\omega t)} \end{cases}. \tag{11}$$

Imposing the Gauss's law $\nabla \cdot \left(\|\varepsilon\| \mathbf{E} \right) = 0$ on $\mathbf{E}_{\mathrm{g}}$, we get

$$\mathbf{E}_{\mathrm{g}}(\mathbf{r},t) = \mathbf{E}_{\mathrm{g}}^0 \cos(\alpha_{\mathrm{g}} z+\varphi) e^{i(\beta x-\omega t)} = \left[\frac{\varepsilon_{\parallel} \alpha_{\mathrm{g}} \sin(\alpha_{\mathrm{g}} z+\varphi)}{\varepsilon_{\perp} i\beta \cos(\alpha_{\mathrm{g}} z+\varphi)} E_{\mathrm{g}z}^0, E_{\mathrm{g}y}^0, E_{\mathrm{g}z}^0 \right] \cos(\alpha_{\mathrm{g}} z+\varphi) e^{i(\beta x-\omega t)} . \tag{12}$$

Using the Faraday's law $\nabla \times \mathbf{E} = i\omega\mu_0 \mathbf{H}$, we can get magnetic fields from Supplementary Equation (11) and (12)

$$\begin{cases} \mathbf{H}_1 = \dfrac{-i}{\omega\mu_0}(\alpha_1 E_{1y}^0, \dfrac{k_0^2\varepsilon_1}{\alpha_1}E_{1x}^0, i\beta E_{1y}^0)e^{-\alpha_1 z}e^{i(\beta x-\omega t)} \\ \mathbf{H}_g = \dfrac{-i}{\omega\mu_0}\left[\alpha_g \sin(\alpha_g z+\varphi)E_{gy}^0, -i\beta\cos(\alpha_g z+\varphi)E_{gz}^0 - \alpha_g\sin(\alpha_g z+\varphi)E_{gx}^0, i\beta\cos(\alpha_g z+\varphi)E_{gy}^0\right]e^{i(\beta x-\omega t)} \\ \mathbf{H}_2 = \dfrac{-i}{\omega\mu_0}(-\alpha_2 E_{2y}^0, -\dfrac{k_0^2\varepsilon_2}{\alpha_2}E_{2x}^0, i\beta E_{2y}^0)e^{\alpha_2(z+d)}e^{i(\beta x-\omega t)} \end{cases} \quad (13)$$

For a TE polarized ordinary waveguide mode, there are three field components (H_x, E_y, H_z). Imposing the interface conditions on $\mathbf{H}_x$ and $\mathbf{E}_y$ at the two interfaces ($z=0,\ z=\text{-}d$), we get

$$\begin{cases} \alpha_1 E_{1y}^0 = \alpha_g \sin\varphi E_{gy}^0 \\ \alpha_g \sin(-\alpha_g d+\varphi)E_{gy}^0 = -\alpha_2 E_{2y}^0 \\ E_{1y}^0 = \cos\varphi E_{gy}^0 \\ \cos(-\alpha_g d+\varphi)E_{gy}^0 = E_{2y}^0 \end{cases}, \quad (14)$$

which can be reformulated into

$$\alpha_g d = \tan^{-1}\left(\frac{\alpha_1}{\alpha_g}\right) + \tan^{-1}\left(\frac{\alpha_2}{\alpha_g}\right) + m\pi \ (\alpha_g = \sqrt{\varepsilon_\perp k_0^2 - \beta^2}). \quad (15)$$

Substituting α_1 α_2 and α_g into Supplementary Equation (15) and let $\beta_o = \beta$, we can get the eigenvalue equation for TE polarized ordinary waveguide modes

$$\sqrt{\varepsilon_\perp k_0^2 - \beta_o^2}\,d = \tan^{-1}\left(\frac{\sqrt{\beta_o^2 - k_0^2\varepsilon_1}}{\sqrt{\varepsilon_\perp k_0^2 - \beta_o^2}}\right) + \tan^{-1}\left(\frac{\sqrt{\beta_o^2 - k_0^2\varepsilon_2}}{\sqrt{\varepsilon_\perp k_0^2 - \beta_o^2}}\right) + m\pi. \quad (16)$$

Similarly, for the TM polarized extraordinary waveguide mode (E_x, H_y, E_z), we have

$$\sqrt{\frac{\varepsilon_\perp}{\varepsilon_\parallel}}\sqrt{\varepsilon_\parallel k_0^2 - \beta_e^2}\,d = \tan^{-1}\left(\frac{\sqrt{\beta_e^2 - k_0^2\varepsilon_1}\,\varepsilon_\perp}{\sqrt{\dfrac{\varepsilon_\perp}{\varepsilon_\parallel}}\sqrt{\varepsilon_\parallel k_0^2 - \beta_e^2}\,\varepsilon_1}\right) + \tan^{-1}\left(\frac{\sqrt{\beta_e^2 - k_0^2\varepsilon_2}\,\varepsilon_\perp}{\sqrt{\dfrac{\varepsilon_\perp}{\varepsilon_\parallel}}\sqrt{\varepsilon_\parallel k_0^2 - \beta_e^2}\,\varepsilon_2}\right) + n\pi, \quad (17)$$

where $\beta_e = \beta$. In Supplementary Equation (16) and (17) m and n are non-negative integers.

Supplementary Note 3: Mode Profiles Normalization and Coupling Factors Calculation

According to Poynting's theorem, the power density in the X direction carried by the ordinary (TE) and extraordinary (TM) waveguide modes can be expressed as

$$P_{\mathrm{o}} = \frac{1}{2} E_y(z) H_z(z) = \frac{\beta_{\mathrm{o}}}{2\omega\mu_0} \int_{-\infty}^{+\infty} E_y^2(z) dz \tag{18}$$

and

$$P_{\mathrm{e}} = -\frac{1}{2} E_z(z) H_y(z) = \frac{k_0^2}{2\omega\mu_0\beta_{\mathrm{e}}} \left[\int_{-\infty}^{-d} \varepsilon_2 E_{2z}^2(z) dz + \int_{-d}^{0} \varepsilon_{\parallel} E_{gz}^2(z) dz + \int_{0}^{+\infty} \varepsilon_1 E_{1z}^2(z) dz \right], \tag{19}$$

respectively. Thus, the ordinary and extraordinary mode profiles can be normalized as

$$\bar{E}_y(z) = \frac{E_y(z)}{\sqrt{P_{\mathrm{o}}}} \tag{20}$$

and

$$\bar{E}_z(z) = \frac{E_z(z)}{\sqrt{P_{\mathrm{e}}}}, \tag{21}$$

respectively. Of course, Supplementary Equation (20) and (21) can be further normalized by dividing the maximum of $\bar{E}_y(z)$ and $\bar{E}_z(z)$—$\max\{\bar{E}_y(z), \bar{E}_z(z)\}$.

If we take the interval $0\ \mathrm{nm} \le z \le 100\ \mathrm{nm}$ to be the efficient coupling region of the waveguide modes and the s-SNOM tip-induced hot spot, the coupling factors can be defined as

$$CF_{\mathrm{o}} = \frac{\int_{0}^{100} \bar{E}_y(z) dz}{\int_{-\infty}^{+\infty} \bar{E}_y(z) dz} \tag{22}$$

and

$$CF_{\mathrm{e}} = \frac{\int_{0}^{100} \bar{E}_z(z) dz}{\int_{-\infty}^{+\infty} \bar{E}_z(z) dz} \tag{23}$$

for the ordinary and extraordinary modes, respectively.

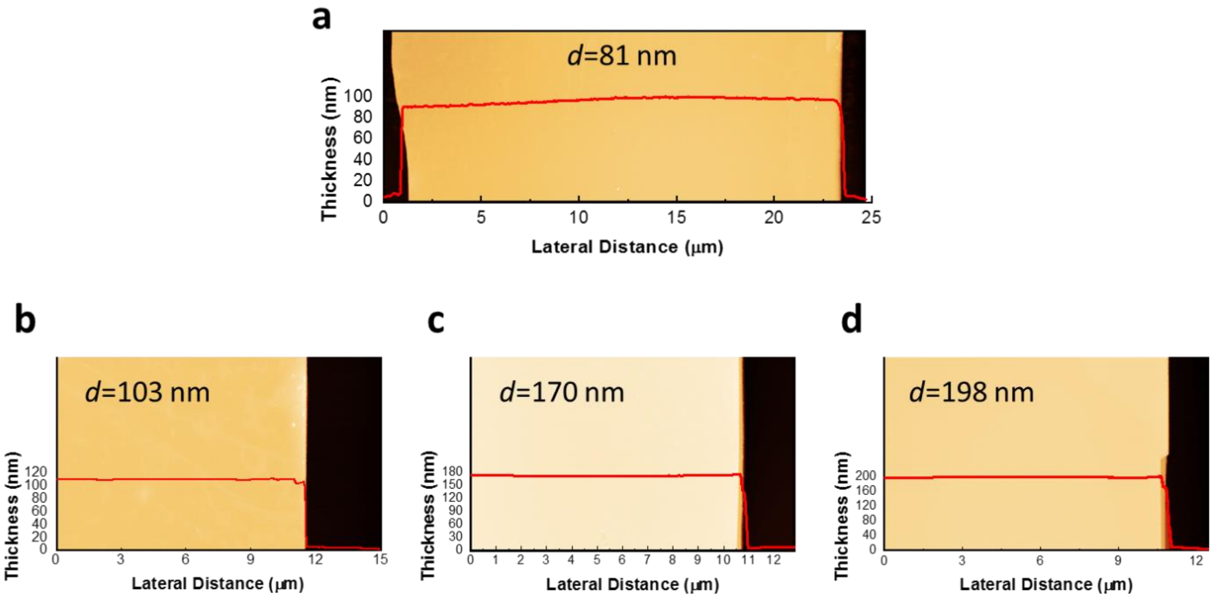

Supplementary Figure 2 | AFM images and height profiles for MoS_2 samples of different thicknesses. (**a**) *d*=81 nm; (**b**) *d*=103 nm; (**c**) *d*=170 nm; (**d**) *d*=198 nm.

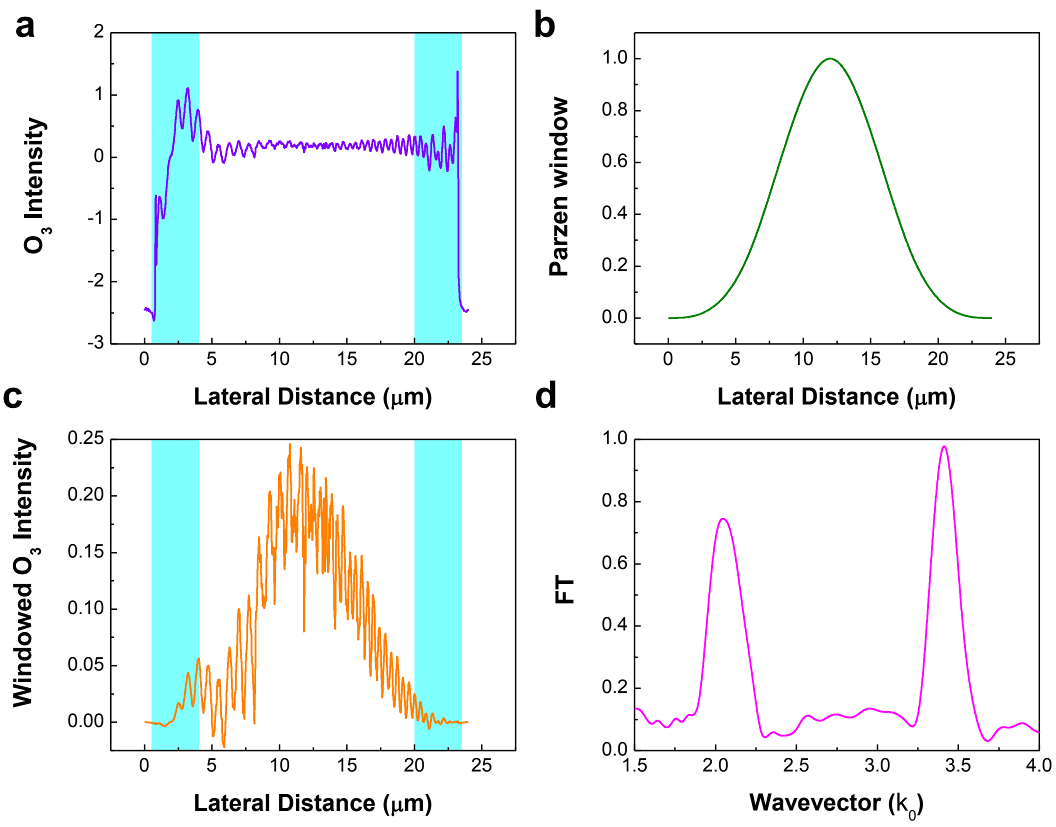

Supplementary Figure 3 | The schematic diagram of using a window function to suppress the edge effect in Fourier transform. (**a**) Subtract the average value of the whole profile from the original data to suppress the DC component in the momentum-space spectra, the shadowed areas indicate where the edge effect exists, widths of the shadows are 3 μm; (**b**) The Parzen window function used to suppress the edge effect; (**c**) The windowed optical profile obtained by multiplying **a** with **b**, the shadowed areas indicate that the edge effect has been effectively suppressed; (**d**) The Fourier transform of **c**, reflecting mainly the spatial frequencies in the center areas of the near-field images.

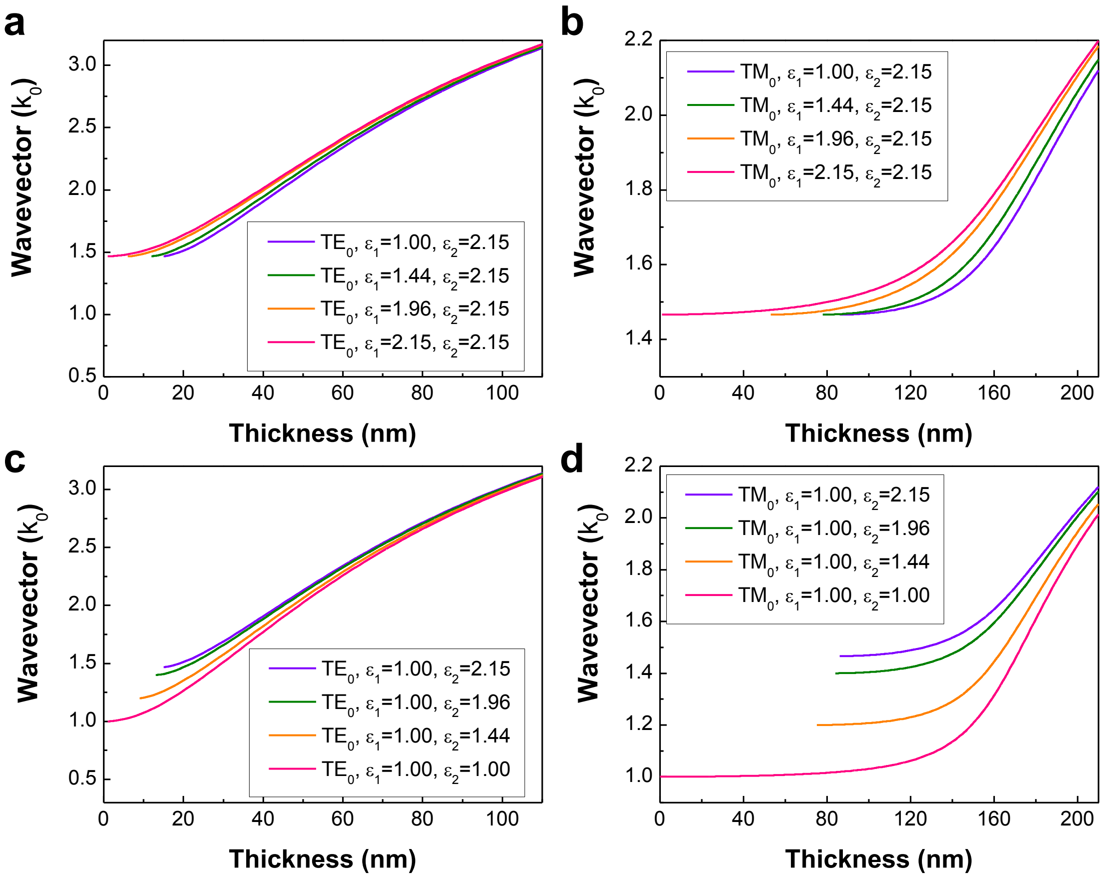

Supplementary Figure 4 | Thickness dispersions of MoS_2 waveguide with different degrees of symmetry. (**a**) and (**b**) Thickness dispersions of the TE_0 and TM_0 mode with increasing superstrate dielectric constant, respectively; (**c**) and (**d**) Thickness dispersions of the TE_0 and TM_0 mode with decreasing substrate dielectric constant, respectively. The cut-off thickness decreases with the increasing degree of symmetry.

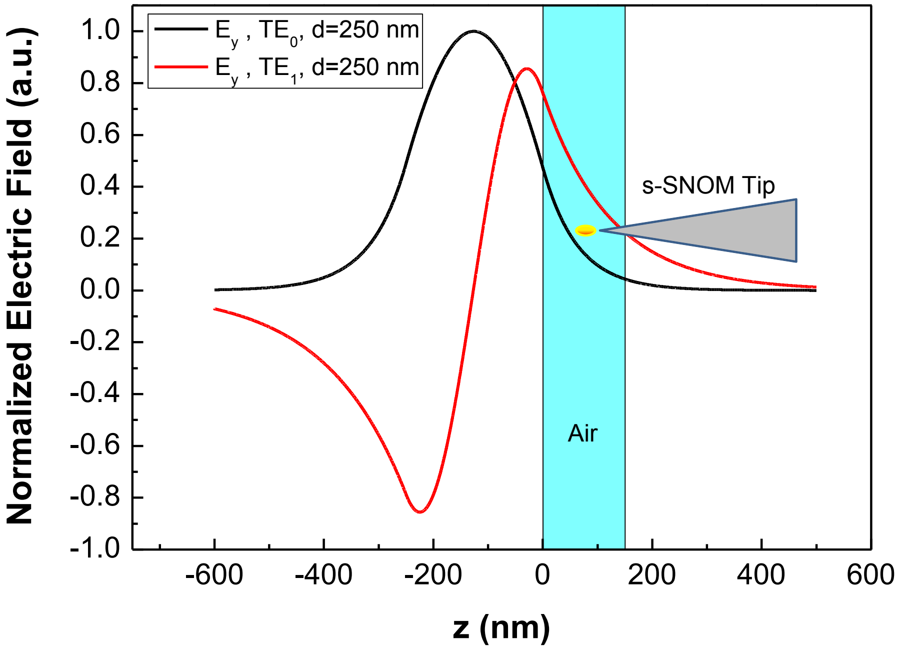

Supplementary Figure 5 | Electric field profiles of the fundamental and the first order TE waveguide modes. The high order modes possess smaller in-plane wavevectors compared with the fundamental modes, thus their evanescent fields extend much further out of the sample surface, leading to high excitation efficiencies of these high order waveguide modes and the enhanced interference visibilities in the s-SNOM images.

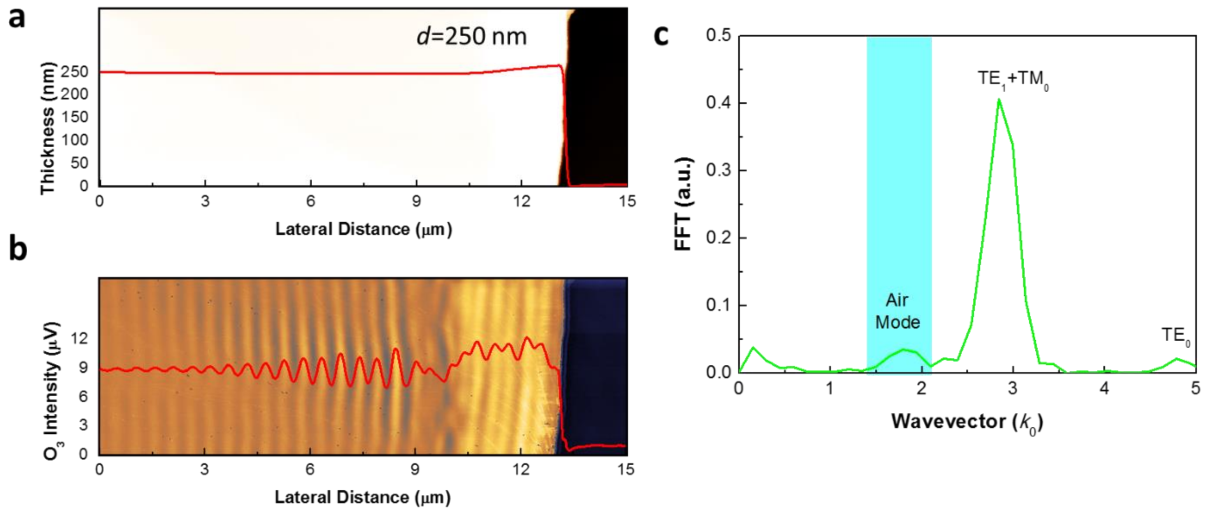

Supplementary Figure 6 | Experimental verification of enhanced interference visibility of TE_1 mode. We used a 250-nm-thick MoS_2 sample supporting the first order TE mode to verify our explanation of the varying interference visibilities in Supplementary Fig 5 and main text. (**a**) AFM image and height profile for a 250-nm-thick MoS_2 sample; (**b**) Near-field image and optical profile of the 250-nm-thick MoS_2 sample; (**c**) Spatial frequency domain spectrum of the optical profile in **b**. Note that the frequency difference between TE_1 and TM_0 is expected to be $0.177k_0$, too small to be resolved in **c**.

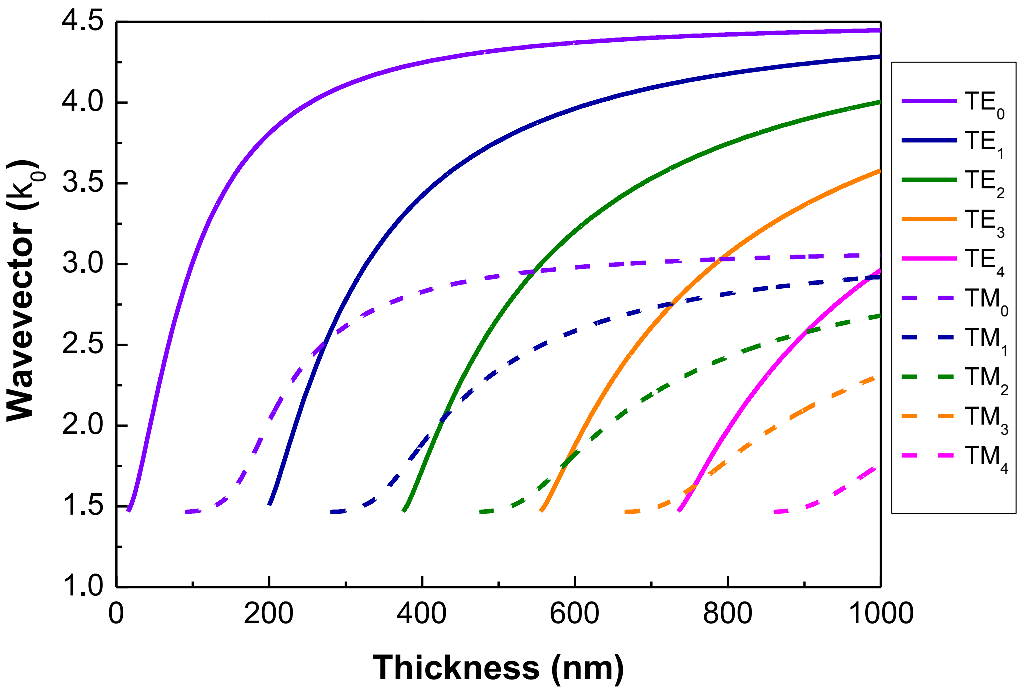

Supplementary Figure 7 | Thickness dispersions of air-MoS_2-SiO_2 waveguide. The MoS_2 thickness range from 0 to 1000 nm. There are five TE modes (m=0~4) and five TM modes (n=0~4) for a waveguide of a 1000-nm-thick MoS_2 guide layer.

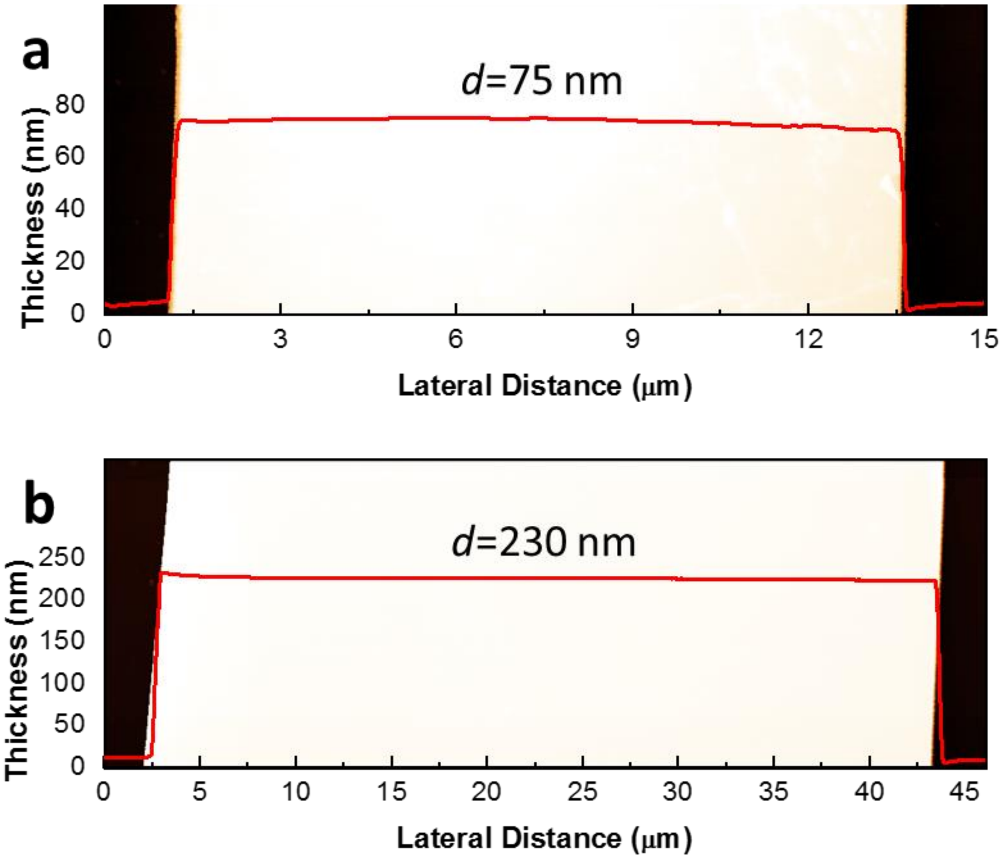

Supplementary Figure 8 | AFM images and height profiles for h-BN samples of different thicknesses. (**a**) *d*=75 nm; (**b**) *d*=230 nm.

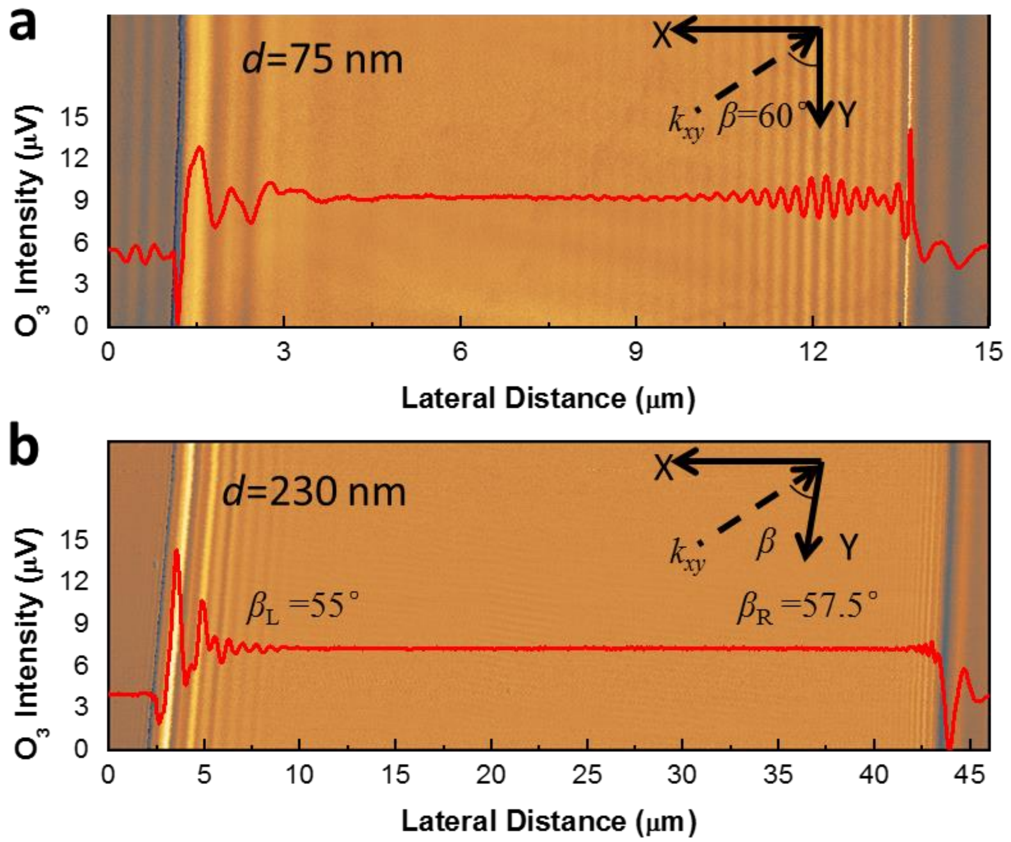

Supplementary Figure 9 | Large-area near-field images and real-space fringe profiles of h-BN samples. (**a**) *d*=75 nm; (**b**) *d*=230 nm.

Supplementary References

1 Born, M. *et al*. *Principles of Optics*; Cambridge University Press: Cambridge, U.K., 1999.
